# Focal ischemic stroke modifies microglia-derived exosomal miRNAs: potential role of mir-212-5p in neuronal protection and functional recovery

**DOI:** 10.1186/s40659-023-00458-x

**Published:** 2023-10-03

**Authors:** Si-si Li, Jia-jia Wu, Xiang-xin Xing, Yu-lin Li, Jie Ma, Yu-jie Duan, Jun-peng Zhang, Chun-lei Shan, Xu-yun Hua, Mou-xiong Zheng, Jian-guang Xu

**Affiliations:** 1 School of Rehabilitation Science, Shanghai University of Traditional Chinese Medicine, NO. 1200, Cailun Road, Shanghai, 201203 Shanghai China; 2 Department of Physical Medicine and Rehabilitation, The Second Affiliated Hospital, Yuying Children’s Hospital of Wenzhou Medical University, Wenzhou, 325027 China; 3 Center of Rehabilitation Medicine, Yueyang Hospital of Integrated Traditional Chinese and Western Medicine, Shanghai University of Traditional Chinese Medicine, Shanghai, 200437 China; 4 Engineering Research Center of Traditional Chinese Medicine Intelligent Rehabilitation, Ministry of Education, Shanghai, China; 5 Department of Traumatology and Orthopedics, Yueyang Hospital of Integrated Traditional Chinese and Western Medicine, Shanghai University of Traditional Chinese Medicine, Shanghai, 200437 China

**Keywords:** Ischemic stroke, Microglial, Exosomal, MiR-212-5p, PlexinA2, Neuronal protection

## Abstract

**Background:**

Ischemic stroke is a severe type of stroke with high disability and mortality rates. In recent years, microglial exosome-derived miRNAs have been shown to be promising candidates for the treatment of ischemic brain injury and exert neuroprotective effects. Mechanisms underlying miRNA dysregulation in ischemic stroke are still being explored. Here, we aimed to verify whether miRNAs derived from exosomes exert effects on functional recovery.

**Methods:**

MiR-212-5p agomir was employed to upregulate miR-212-5p expression in a rat model of middle cerebral artery occlusion/reperfusion (MCAO/R) as well as an oxygen-glucose deprivation/reoxygenation (OGD/R) in vitro. Western blot analysis, qRT–PCR and immunofluorescence staining and other methods were applied to explore the underlying mechanisms of action of miR-212-5p.

**Results:**

The results of our study found that intervention with miR-212-5p agomir effectively decreased infarct volume and restored motor function in MCAO/R rats. Mechanistically, miR-212-5p agomir significantly reduced the expression of PlexinA2 (PLXNA2). Additionally, the results obtained in vitro were similar to those achieved in vivo.

**Conclusion:**

In conclusion, the present study indicated that PLXNA2 may be a target gene of miR-212-5p, and miR-212-5p has great potential as a target for the treatment and diagnosis of ischemic stroke.

## Introduction

Stroke is a common cerebrovascular emergency that leads to serious brain tissue damage and is attributed to a sudden occlusion or rupture of blood vessels that prevent blood flow to the brain [1]. Two main stroke types have been identified: hemorrhagic stroke and ischemic stroke [2]. Ischemic stroke is characterised by high incidence, mortality, disability and recurrence rates, accounting for approximately 80% of all strokes [3]. Brain damage following permanent or transient focal cerebral ischemia triggers a cascade of pathophysiological events, including free radical release, microglial polarization, blood–brain barrier (BBB) dysfunction, neuronal apoptosis and neuroinflammation [4]. The ischemic penumbra exhibits an increase in neuronal apoptosis at the acute stage of cerebral ischemia, and this process is believed to constitute an important part of cerebral ischemic damage [5]. Salvaging the penumbra is central to the concept of neuroprotection from ischemic stroke [6].

In the central nervous system (CNS), microglia function as resident immune cells and have dual functions in brain tissue injury, regeneration and repair following ischemic stroke [7]. Microglia are categorised into proinflammatory M1 and anti-inflammatory M2 subtypes [8]. M2 microglia are presumed to perform neuroprotective functions by augmenting axonal regeneration, remyelination, neurogenesis, and angiogenesis [9]. Exosomes are derived from the internal vesicles of multivesicular bodies with a diameter of 30–150 nm and are secreted by different cell types within the brain, including microglia, neurons, and astrocytes [10]. As soon as the multivesicular body fuses with the plasma membranes, exosomes are released into the extracellular space, while protecting their contents from degradation [11, 12]. They are involved in the transport of functional biochemicals, such as messenger RNA (mRNA), microRNAs (miRNAs), cytokines, and proteins, and, as a result, participate in intercellular communication by directly transferring genetic material to target cells [13].

A recent study revealed that exosomal miRNAs exhibit regulatory effects on target cells and thus may in fact represent a novel pathway of intracellular communication [14]. Notably, miRNAs, which are small noncoding RNA molecules, are processed to form ribonucleoprotein complexes that bind to target mRNA, leading to mRNA degradation or translational inhibition [15]. One miRNA can bind to multiple targets and simultaneously functionally inhibit multiple mRNAs to participate in biological processes, thereby achieving therapeutic effects on diseases [16]. Based on accumulating evidence, miRNAs have vital functions in a broad range of neurological diseases, such as spinal cord injury [17], traumatic brain injury [18], and stroke [19]. They are mediators that are essential for the endogenous neuroprotective response to ischemic preconditioning in the brain [20]. Moreover, miRNAs play critical roles in the establishment of functional neurons by regulating neuronal morphology [21]. Some miRNAs are highly abundant in brain, where they serve as effectors required for the development and maintenance of neuronal phenotypes [22]. They are also expressed in dendrites, where they have been implicated in synaptic plasticity by regulating synaptic and dendritic spine structures [23]. Among these miRNAs, miR-212-5p is expressed at higher levels in a normal brain but shows a significant reduction after ischemic stroke. However, the mechanism of action of miR-212-5p in mediating the crosstalk between neurons and microglial cells is still poorly understood.

Further researches are still needed to determine whether miR-212-5p exerts a neuroprotective effect after cerebral ischemia, as well as the molecular mechanisms involved in conferring the neuroprotective properties of miR-212-5p. This study aims to verify whether miR-212-5p is a potential neuroprotective agent for the treatment of ischemic stroke. There was also an investigation into potential miR-212-5p targets. We identified that Plexin A2 (PLXNA2) was the target gene of exosomal miR-212-5p through online databases. We hypothesised that miR-212-5p functions as a neuroprotective miRNA and inhibits neurological damage by selectively targeting PLXNA2 to facilitate neuroprotection and functional recovery following ischemic brain injury.

## Results

### Cytokine production by microglia after stroke

**Fig. 1:**
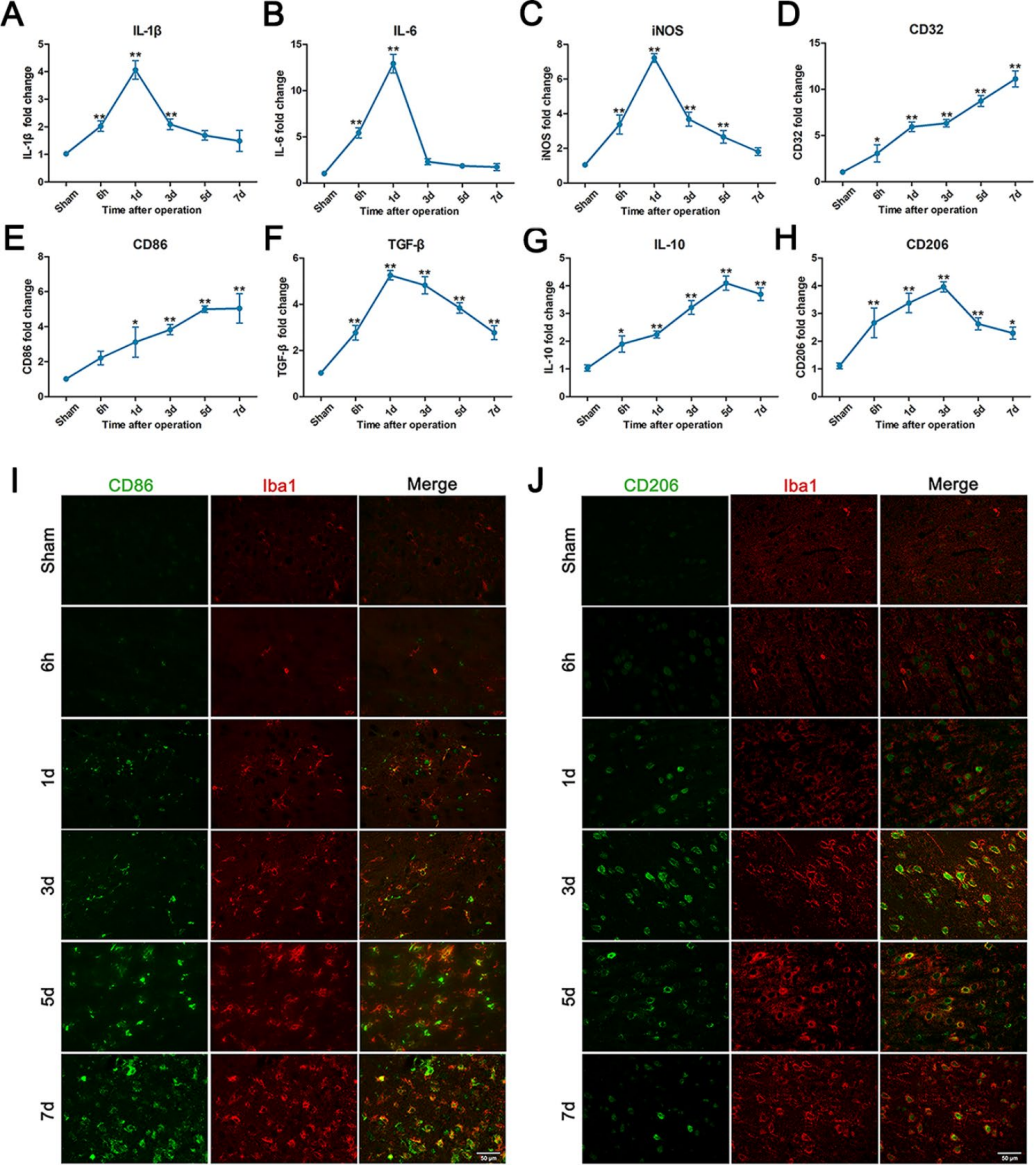
Measurement of pro- and anti‐inflammatory cytokine levels at 6 h, 1, 3, 5 and 7 days postsurgery. **A-E** Proinflammatory cytokines released by microglia (IL‐1β, IL-6, INOS, CD32 and CD86). **F-H** Anti-inflammatory cytokines released by microglia (anti‐inflammatory cytokines TGF-β, IL‐10 and CD206). **I** Double immunofluorescence staining was applied to examine the expression of CD86 (in green) and Iba1 (in red) in the ischemic penumbra of the cortex at different time points. **J** Double immunofluorescence staining was performed to examine the expression of CD206 (in green) and Iba1 (in red) at different time points. Scale bar = 50 μm. The data are presented as the means ± SEM (n = 5 per group). *P < 0.05 and **P < 0.01 compared with the sham group

The time course of changes in microglial phenotypes in the cortex ischemic penumbra region following transient cerebral ischemia was quantified by measuring the intracellular levels of pro- and anti‐inflammatory cytokines using qRT–PCR. The mRNA expression of M1-related proinflammatory mediators, including IL-6, IL-1β and iNOS, was obviously and progressively increased after cerebral ischemia, reaching a peak at 24 h (Fig. 1A-C). The expression of CD32 and CD86 increased gradually after ischemia and remained elevated for at least 7 days (Fig. 1D and E). Regarding M2 markers, including TGF‑β, IL-10, and CD206, we found that TGF-β expression peaked at 24 h after surgery in the present study (Fig. 1F). IL-10 and CD206 were induced after MCAO/R and peaked at 3 to 5 days postinjury (Fig. 1G, H). Dual immunofluorescence staining for CD86/Iba1 and CD206/Iba1 in the ischemic penumbra of the cerebral cortex showed that CD86 expression increased gradually over time for at least 7 days (Fig. 1I). Additionally, CD206 expression was remarkably lower on day 7 than on days 3 and 5 (Fig. 1J). Therefore, the expression of M2 microglial markers was abundant at day 3 after surgery.

### The expression of miR-212-5p is decreased in microglial exosomes from the ischemic penumbra at 3 days after MCAO/R

**Fig. 2:**
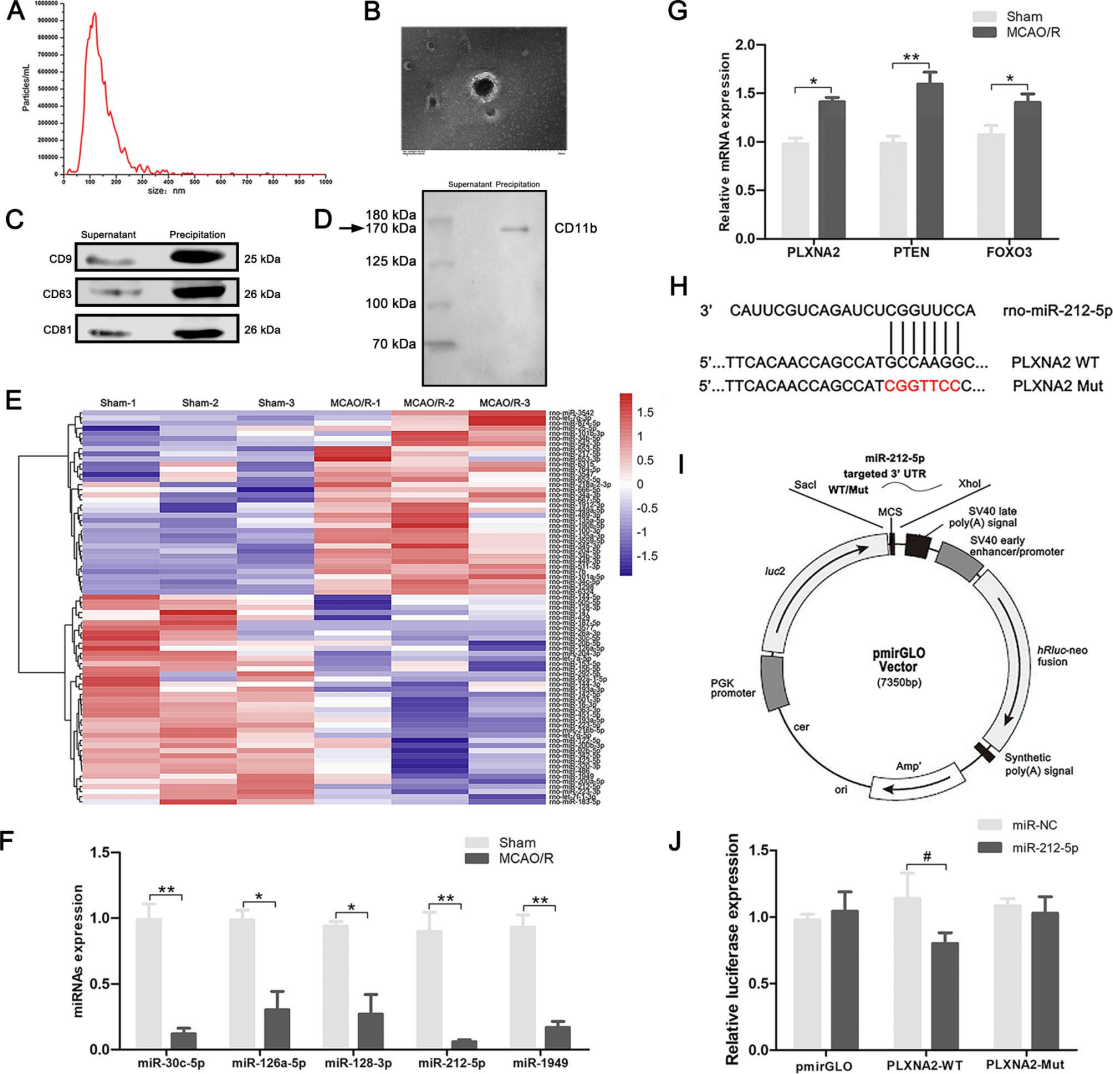
MiRNA sequencing of microglial exosomes 3 days after MCAO/R and qRT–PCR validation. **A, B** Characterisation of microglial exosomes using nanoparticle tracking analysis and transmission electron microscopy scanning. Scale bar = 200 nm. **C** The exosome markers CD9, CD63, and CD81 were detected using western blot analysis. **D** Microglia-derived exosomes were detected by western blot analysis using the microglia marker CD11b. **E** Heatmap showing the levels of miRNAs in microglial exosomes at 3 days after MCAO/R. **F** Expression of miR-30c-5p, miR-126a-5p, miR-128-3p, miR-212-5p and miR-1949 in the ischemic penumbra of the cortex at 3 days after MCAO/R was determined using qRT–PCR. **G** Expression levels of the miRNA-targeting genes PLXNA2, PTEN and FOXO3 in the ischemic penumbra of the cortex at 3 days after MCAO/R. **H** The target sites of miR-212-5p in PLXNA2 mRNA 3’ untranslated region (3’UTR). **I** Map of the pmirGLO luciferase reporter vector. **J** Dual luciferase assays revealed the binding of miR-212-5p to the 3’UTR of PLXNA2. The data are presented as the means ± SEM (n = 5 per group). *P < 0.05 and **P < 0.01 compared with the sham group. ^#^*P* < 0.05 compared with the miR-NC group

3 days after MCAO/R, microglial exosomes were collected from the ischemic penumbral cortex to further probe the roles of microglial exosomal miRNAs in the pathological changes after ischemic stroke. The isolated exosome samples were characterised using NTA, TEM, and western blot analysis of the exosomal surface markers (CD9, CD63, CD81). As shown in Fig. 2A, B, exosomes exhibited a typical cup-shaped membrane vesicle morphology with a diameter ranging from approximately 30–120 nm. Exosome-specific markers CD9, CD63, and CD81 were all present at high levels in the precipitate (Fig. 2C). Western blot analysis also revealed high expression of CD11b in precipitate (Fig. 2D). The expression levels of miRNAs in microglial exosomes was determined using miRNA sequencing in sham and MCAO/R rats. The heatmap of miRNA expression is shown in Fig. 2E. The altered expression of miRNAs was further validated using qRT–PCR. We screened miR-30c-5p, miR-126a-5p, miR-128-3p, miR-212-5p and miR-1949, which are reportedly responsible for neurodegeneration. The experimental results showed that the miRNAs were significantly downregulated in the ischemic penumbra (Fig. 2F). Based on our miRNA sequencing and qRT–PCR results, significantly reduced expression of miR-212-5p was observed in the ischemic penumbra and it was selected for further study. A search of prediction databases identified several predicted mRNA targets of miR-212-5p that may be involved in the neuroprotective effects of the miR-212-5p. The expression levels of PLXNA2, PTEN and FOXO3 was detected in the ischemic penumbra (Fig. 2G). Then, a dual-luciferase reporter system was carried out to validate PLXNA2 as a direct target of miR-212-5p. Transfection of miR-212-5p significantly decreased the luciferase activity in PC12 cells transfected with the wild-type 3′-UTR of PLXNA2 but did not inhibit luciferase activity in cells containing the mutant construct, indicating that PLXNA2 was a direct target of miR-212-5p (Fig. 2H-J).

### MiR-212-5p improved motor functional recovery and attenuated brain damage

**Fig. 3:**
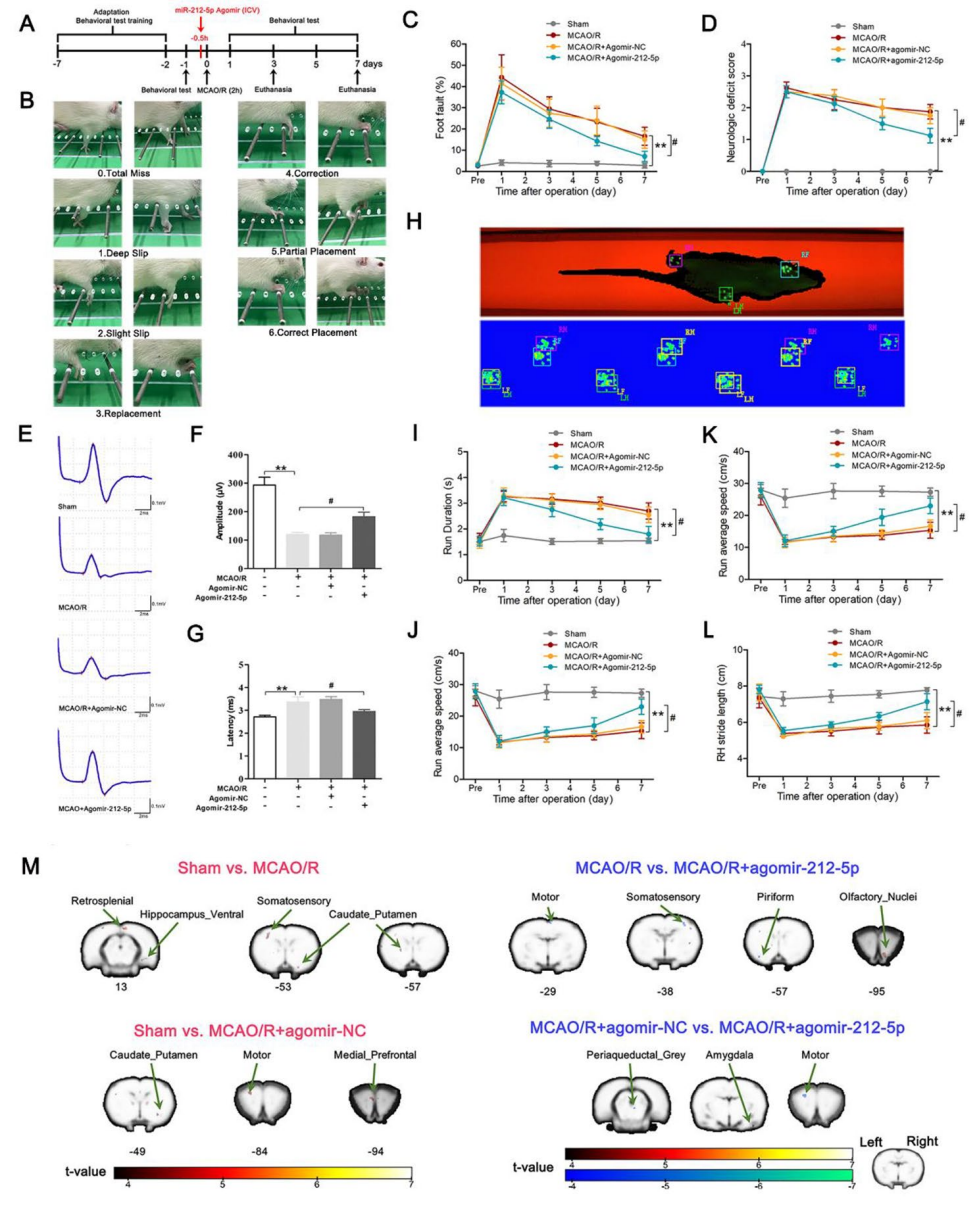
Treatment with agomir-212-5p decreased neurological deficits and improved locomotor activity 7 days after MCAO/R. **A** The flow chart of the experiment. **B** Schematic of the scoring criteria for the foot fault test. **C** Foot fault test results. **D** Neurological deficit scores were evaluated using the Zea Longa 5-point scheme. **E** Illustrative waveforms of motor evoked potential (MEP) for each group. **F** The amplitude of MEP for each group. **G** The latency of MEP for each group. n = 6 per group. **H** Schematic representation of the CatWalk gait analysis. **I** Running duration (s). **J** Average running speed (cm/s). **K** Right forelimb stride length (cm). **L** Right hindlimb stride length (cm). The data are presented as the means ± SEM (n = 8 per group). **M** Results of functional connectivity analysis among four groups. *P < 0.05 and **P < 0.01 compared with the sham group, ^#^*P* < 0.05 and ^##^*P* < 0.01 compared with the MCAO/R group

Figure 3 A illustrated the flow chart of the experiment. We performed behavioural tests to explore whether miR-212-5p improved the motor function of MCAO/R rats. The foot fault test was assessed using an irregularly spaced horizontal ladder (Fig. 3B). After MCAO/R, the misstep rate of right side was significantly higher compared to the sham group, while the rate of misstep in the MCAO/R + agomir-212-5p group was significantly lower than that in the MCAO/R group on postoperative day 7 (Fig. 3C). MCAO/R rats revealed higher neurological deficit scores, while MCAO/R + agomir-212-5p group showed a lower neurological deficit score compared with the MCAO/R group (Fig. 3D). After MCAO/R, animals showed prolonged MEP latencies and reduced peak amplitudes. Animals in the MCAO/R + agomir-212-5p group showed significantly better recovery 7 days after MCAO/R (Fig. 3E-G). In addition, we conducted a CatWalk gait analysis to evaluate motor function (Fig. 3H). The average running speed became slower with longer durations of running after MCAO/R surgery. Agomir-212-5p reduced the running duration to some extent (Fig. 3I); at the same time, it also increased the average running speed (Fig. 3J). The stride length was significantly decreased in the affected limbs at all measured time points after MCAO/R. The rats treated with miR-212-5p exhibited a significantly improved stride length compared with MCAO/R rats (Fig. 3K, L). The results of FC analysis showed that compared with sham group, the FC between the motor cortex (left) and the retrosplenial cortex (right), somatosensory cortex (left), caudate putamen (left and right), the ventral part of hippocampus (right) was decreased in MCAO/R group. In addition, the FC between the motor cortex (left) and medial prefrontal cortex (left), motor cortex (left), caudate putamen (right) was decreased in MCAO/R + agomir-NC group. However, compared with the MCAO/R group, the FC between the motor cortex (left) and somatosensory cortex (right), motor cortex (right), piriform cortex (left) was significantly increased in the MCAO/R + agomir-212-5p group. Meanwhile, compared with the MCAO/R + agomir-NC group, the FC between the motor cortex (left) and periaqueductal grey (right), motor cortex (left) was significantly increased in the MCAO/R + agomir-212-5p group (Fig. 3M; Table 1). Taken together, treatment with agomir-212-5p led to a partial recovery of MEP, enhanced the connection of brain neural network by activating functional connections between the affected motor cortex and sensory cortex, motor cortex in the whole brain, and significantly improved locomotor behaviour.

**Table 1:** Primers sequence for quantitative real-time Polymerase Chain Reaction

Gene	Forward primer (5′-3′)	Reverse primer (5′-3′)
*IL-1β*	TGTTTCCCTCCCTGCCTCTGAC	CGACAATGCTGCCTCGTGACC
*IL-6*	ACTTCCAGCCAGTTGCCTTCTTG	TGGTCTGTTGTGGGTGGTATCCTC
*iNOS*	GAGACGCACAGGCAGAGGTTG	AGCAGGCACACGCAATGATGG
*CD32*	ACACTGTGACACTGATGTGCGAAG	TGTAGTTGGCTTGGGCTTGATGC
*CD86*	TTTCGCAGCCCCAGTTTGATCG	GACCAGCAGAAAGAGACAGCACAG
*TGF-β*	GACCGCAACAACGCAATCTATGAC	CTGGCACTGCTTCCCGAATGTC
*IL-10*	GAACCACCCGGCATCTACTG	TTCCAAGGAGTTGCTCCCGT
*CD206*	TGGACAGACGGACGAGGAGTTC	GCCACCAATCACAACAACACAGTC
*PLXNA2*	ACCGACTGGGCAAGGACTCAC	GCGAGGTAGGCGTTCATATCTTGG
*PTEN*	TTGAAGACCATAACCCACCACAGC	CATTACACCAGTCCGTCCTTTCCC
*FOXO3*	TGGACGCCTGGACCGACTTC	GCTCCGTGCTCGCCAAGATG
*RhoA*	AAGTGGACGGGAAGCAGGTAGAG	CATCAGTGTCTGGGTAGGAGAGAGG
*ROCK2*	GGGAGGTACGACTTGGAAGAAATGG	GCTGCTGTCTATGTCACTGCTGAG
*Nogo-A*	TGCAGTGTTGATGTGGGTGTT	ATCTGCACCTGATGCCGTTC
*NgR*	TCGGAAGGAGCAGGACTCAGAAC	TGAGGGAGGCATAGGATTGGACAG
*β-actin*	TGGCTCTATCCTGGCCTCAC	CGCAGCTCAGTAACAGTCCG

**Fig. 4:**
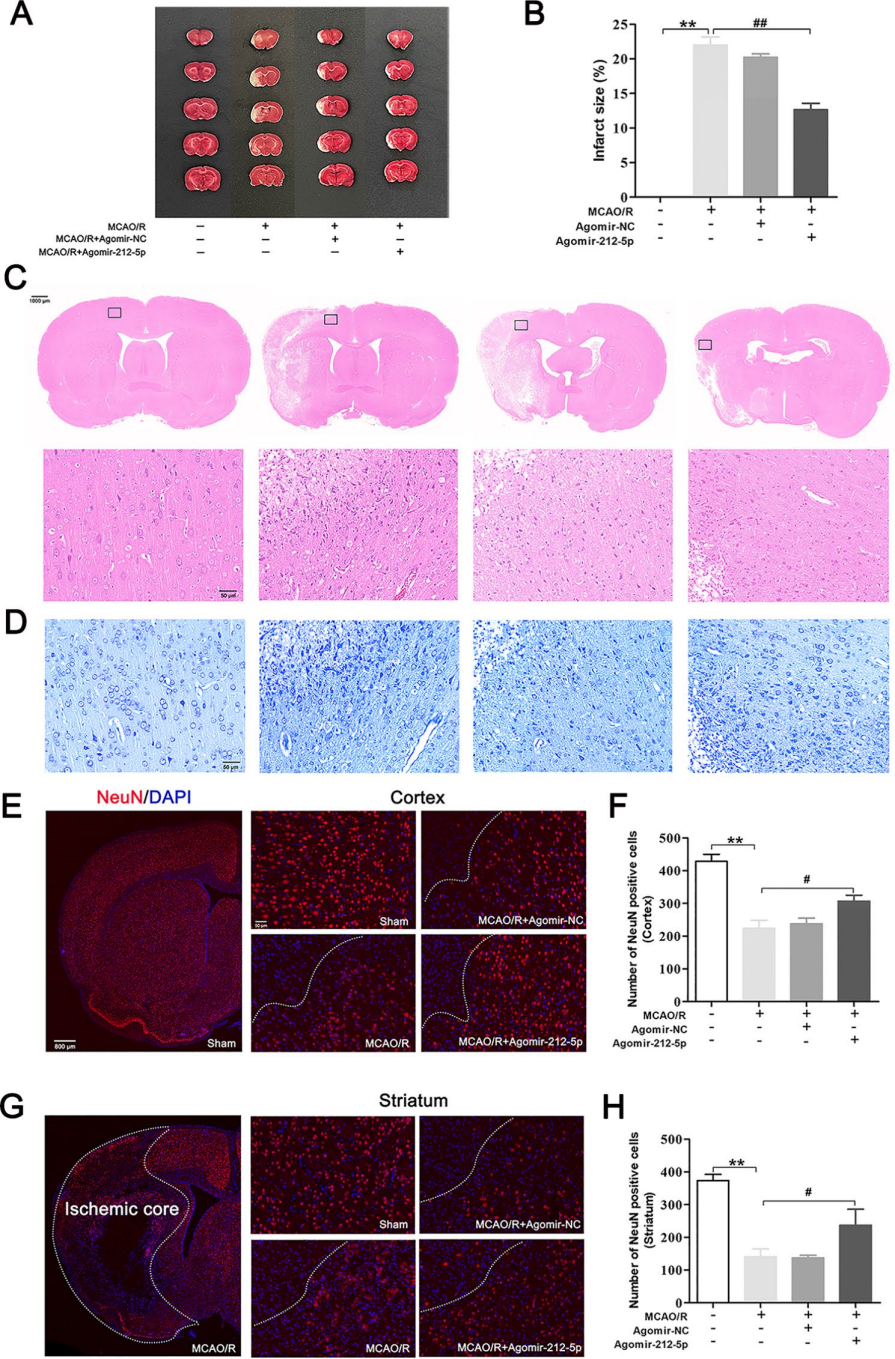
Agomir-212-5p reduces the infarct volume and mitigates neuronal apoptosis 7 days after MCAO/R. **A** Brain sections were stained with TTC to visualise the ischemic lesions at 7 days after MCAO/R. The white area shows the infarct core. **B** Quantitative analysis of the percentage of the infarct area. **C** H&E staining. Scale bars = 1000 μm (low magnification) and 50 μm (high magnification). **D** Nissl staining. Scale bars = 50 μm. **E, F** Immunofluorescence staining for NeuN showing the survival of neurons in the peri-infarct cortex 7 days after MCAO/R. **G, H** The numbers of NeuN + viable neurons in the peri-infarct striatum. The data are presented as the means ± SEM (n = 3 per group). *P < 0.05 and **P < 0.01 compared with the sham group, ^#^*P* < 0.05 and ^##^*P* < 0.01 compared with the MCAO/R group

We collected rat brains on day 7 after MCAO/R and performed TTC staining to test the effects of treatment with agomir-212-5p on the infarct volume (Fig. 4A). TTC staining illustrated a significant increase in white matter was observed in the MCAO/R group, whereas the administration of agomir-212-5p obviously reduced the infarct volume of rats subjected to MCAO/R (Fig. 4B). This change may suggest a neuroprotective effect of agomir-212-5p. Tissue sections were stained with H&E and Nissl to reveal the histological appearance and histological changes in neurons and to further confirm the neuroprotective role of miR-212-5p. H&E staining revealed a disordered cell arrangement, interstitial oedema and a large number of necrotic cells in MCAO/R rats (Fig. 4C). In addition, Nissl staining showed the number of Nissl-positive bodies in the ischemic penumbra of the MCAO/R group was reduced. Agomir-212-5p treatment effectively ameliorated brain tissue loss and neuronal damage (Fig. 4D). NeuN staining further indicated that neuronal apoptosis was decreased after treated with agomir-212-5p in the cortex (Fig. 4E, F) and striatum (Fig. 4G, H) compared with the MCAO/R group. Altogether, these results conclusively showed that miR-212-5p effectively alleviated cerebral ischemia-induced brain damage.

### MiR-212-5p promotes neuroprotection by targeting PLXNA2 in MCAO/R rats

**Fig. 5:**
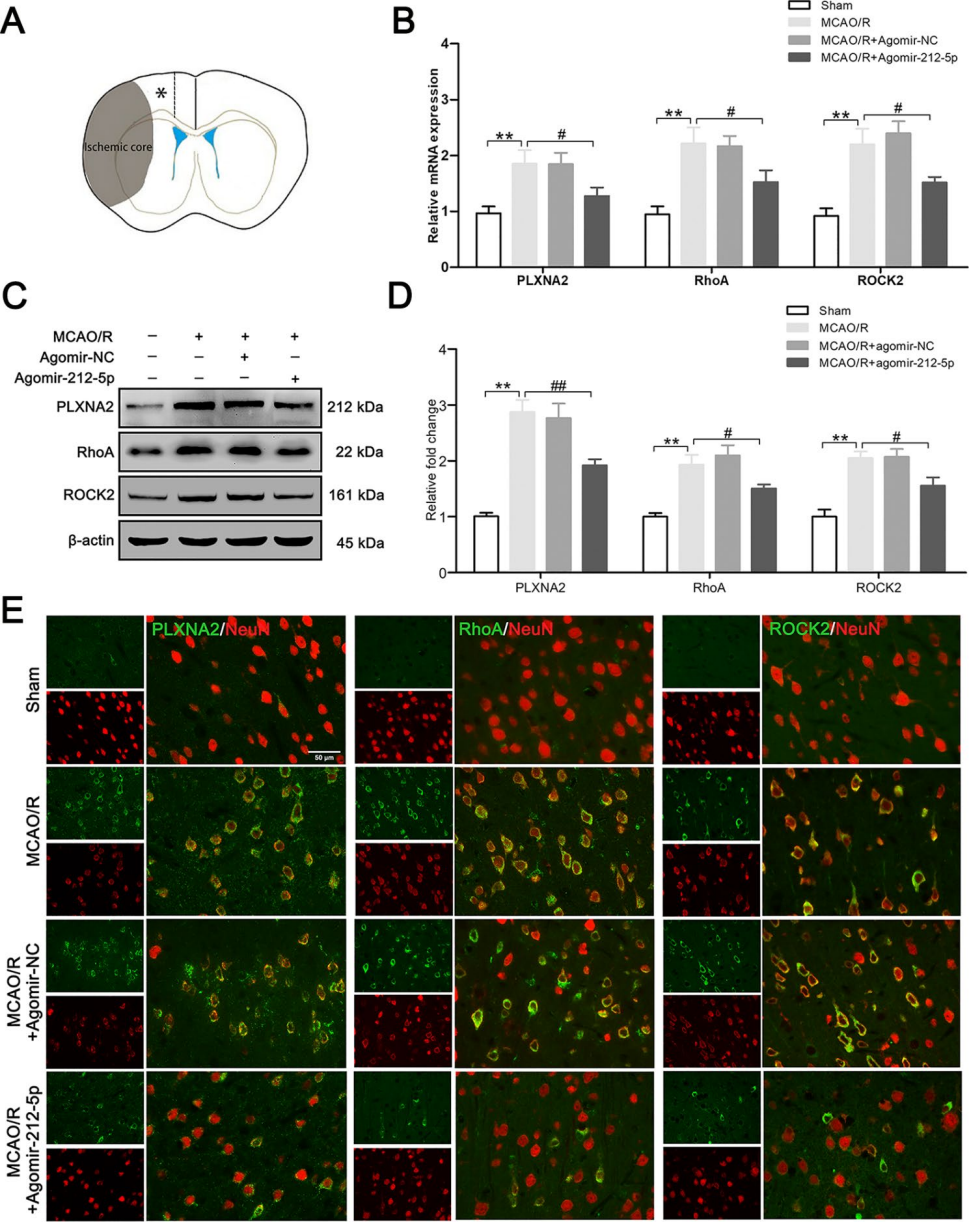
Agomir-212-5p inhibited the PLXNA2 and RhoA/ROCK2 in the ischemic penumbra at 3 days following MCAO/R. **A** The asterisks denote the observation site. **B** PLXNA2, RhoA and ROCK2 mRNA expression in the ipsilateral brain was analysed using qRT–PCR. **C** Representative images of western blots for PLXNA2, RhoA and ROCK2 in each group. β-Actin was detected as an internal reference. **D** Quantitative analysis of the western blot results. **E** Immunofluorescence staining for the PLXNA2, RhoA and ROCK2 proteins in neurons from each group. Scale bar = 50 μm. The data are presented as the means ± SEM (n = 4–6 per group). *P < 0.05 and **P < 0.01 compared with the sham group, ^#^*P* < 0.05 and ^##^*P* < 0.01 compared with the MCAO/R group

Further investigation of the mechanism underlying the neuroprotective effects of miR-212-5p was achieved by detecting the expression of the miR-212-5p target gene PLXNA2. At 3 days after MCAO/R, qRT-PCR analysis showed significantly higher levels of PLXNA2, RhoA, and ROCK2, whereas treatment with agomir-212-5p significantly downregulated their expression in the ischemic penumbra (Fig. 5B). Western blot results were consistent with the qRT–PCR results (Fig. 5C, D). Finally, we examined PLXNA2, RhoA and ROCK2 expression in neurons by performing double immunofluorescence staining, and the neurons in the ischemic penumbra were labelled with a NeuN antibody. Immunofluorescence staining showed that agomir-212-5p attenuated PLXNA2, RhoA and ROCK2 expression in neurons 3 days after MCAO/R (Fig. 5E). The results suggested that PLXNA2 and RhoA/ROCK2 may be involved in the neuroprotective effect of miR-212-5p.

### MiR-212-5p promotes synaptic plasticity and attenuates axonal degeneration in MCAO/R rats

**Fig. 6:**
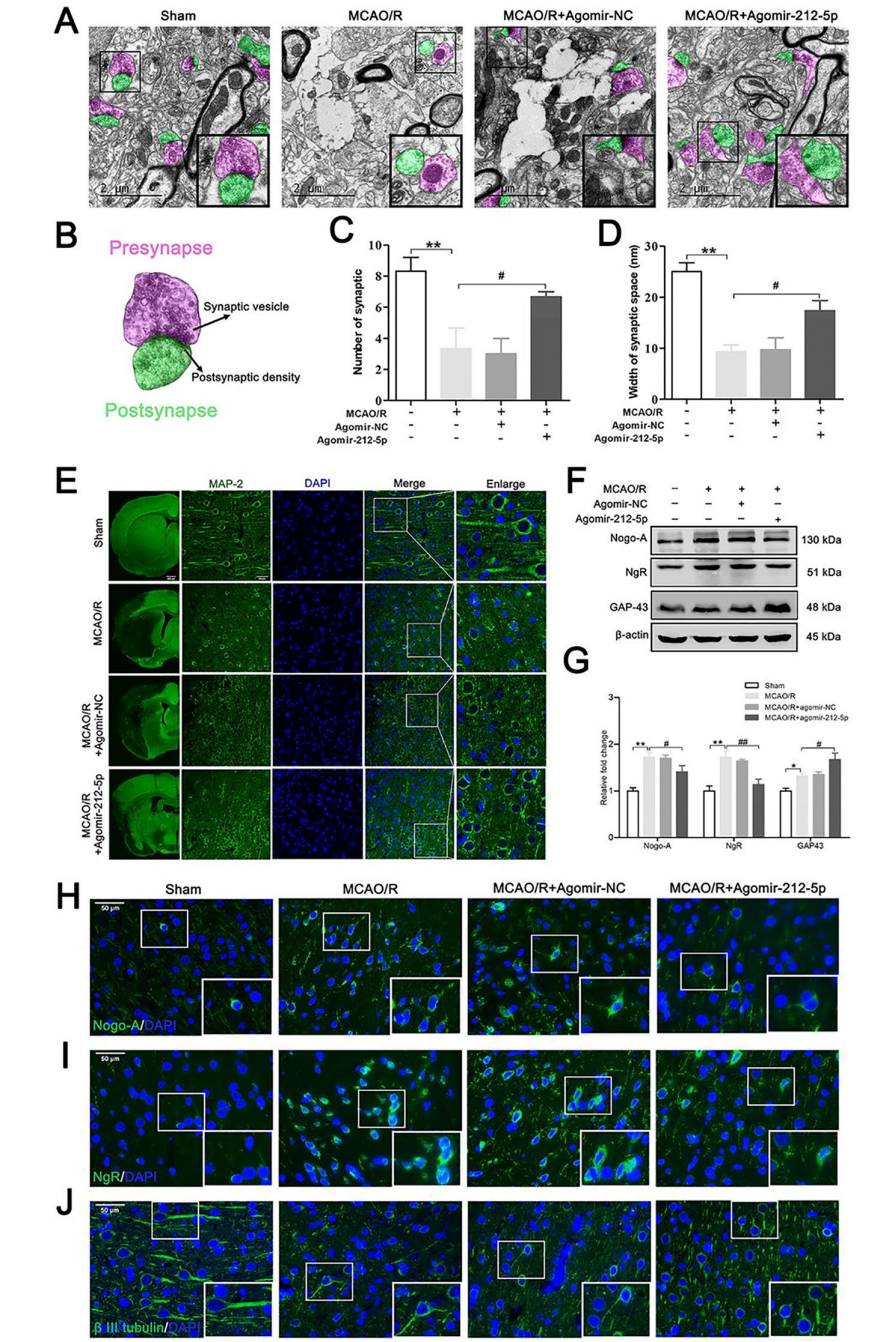
MiR-212-5p promotes synaptic plasticity and attenuates axon degeneration at 7 days following MCAO/R. **A** Ultrastructures of the synapses in the ischemic penumbra analysed using TEM. Scale bar = 2 μm. **B** Schematic of the presynaptic (violet) and postsynaptic (green) structures. **C** The number of synapses in the ischemic penumbra of the cortex in each group. **D** Width of the synaptic space (nm). n = 3 per group. **E** Immunofluorescence staining shows MAP-2 (in green) expression in the ischemic penumbra of the cortex. Nuclei were stained with DAPI and are visualised in blue. Scale bar = 50 μm. **F** Representative images of western blots for Nogo-A, NgR, and GAP-43. **G** Quantitative analysis of the western blot results. n = 4–6 per group. **H-J** Immunofluorescence staining was performed to show the presence of Nogo-A, NgR and β III tubulin in the ischemic penumbra of the cortex. Nuclei were stained with DAPI and are visualised in blue. Scale bar =, 50 μm. The data are presented as the means ± SEM. *P < 0.05 and **P < 0.01 compared with the sham group, ^#^*P* < 0.05 and ^##^*P* < 0.01 compared with the MCAO/R group

Synapse structures in the sham group were complete, including intact pre- and postsynaptic structures. Fewer synaptic vesicles, blurred or missing synaptic clefts and abnormal synaptic structures were observed after MCAO/R surgery (Fig. 6A). Pre- and postsynaptic structures are indicated in light pale violet and green, respectively (Fig. 6B). The number of synapses and width of the synaptic space were improved in response to treatment with agomir-212-5p (Fig. 6C, D). A substantial reduction in the density of MAP-2 staining was observed, however, this phenomenon could be reversed by agomir-212-5p treatment (Fig. 6E). We next examined the effect of agomir-212-5p on attenuating axonal degeneration. The western blot results revealed that the levels of Nogo-A and the NgR, which showed increased expression following MCAO/R, were reduced after intervention with agomir-212-5p. Growth-associated protein-43 (GAP-43), a neuronal-specific protein, was significantly higher in the MCAO/R + agomir-212-5p group than the MCAO/R group (Fig. 6F, G). Western blot analyses of Nogo-A and NgR were further verified by performing immunofluorescence staining (Fig. 6H, I). Treatment with agomir-212-5p strongly protected axons from MCAO/R-induced degeneration (Fig. 6J). Overall, these findings demonstrated that increasing miR-212-5p expression may be a potential strategy for promoting synaptic plasticity and inhibiting axonal degeneration after MCAO/R-induced focal cerebral ischemia.

### MiR-212-5p facilitates dendritic spine formation and dendritic growth in rats after MCAO/R

**Fig. 7:**
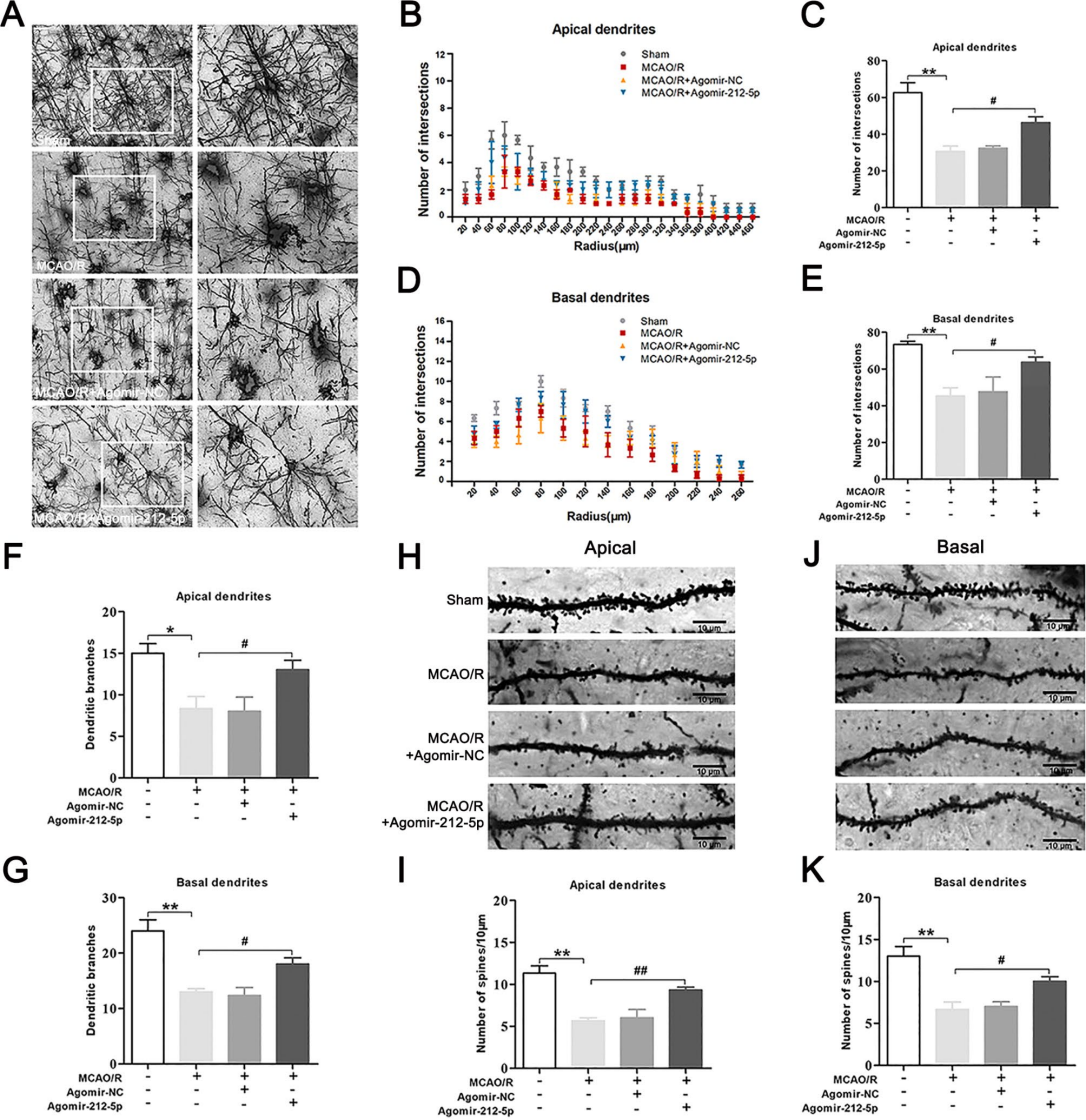
MiR-212-5p promoted dendritic spine formation and dendritic growth at 7 days following MCAO/R. **A** Representative Golgi-Cox-stained photomicrograph of brains from rats subjected to MCAO/R. Scale bar = 50 μm and 20 μm. **B** The apical dendritic intersections were counted at increasing distances from the centre of the soma. **C** The total number of intersections in the apical dendrites. **D** The basal dendritic intersections were counted at increasing distances from the centre of the soma. **E** The total number of intersections for the basal dendrites. **F** The total number of branches of apical dendrites. **G** The total number of branches of basal dendrites. **H** Graphs of dendritic spines on apical dendrites. Scale bar = 10 μm. **I** Density of dendritic spines along apical dendrites. **J** Graphs of dendritic spines on basal dendrites. Scale bar = 10 μm. **K** Density of dendritic spines along basal dendrites. The data are presented as the means ± SEM (n = 3 per group). *P < 0.05 and **P < 0.01 compared with the sham group, ^#^*P* < 0.05 and ^##^*P* < 0.01 compared with the MCAO/R group

We measured dendritic complexity in vivo by performing Golgi-Cox staining of brain sections to assess the role of miR-212-5p. The apical and basal dendrites exhibited marked decreases in complexity at 7 days after MCAO/R, and MCAO/R rats showed a reduced number of dendrite intersections (Fig. 7A-E) and decreased dendritic branches (Fig. 7F, G). We also compared spine densities on apical dendrites and basal dendrites. A significant reduction in dendritic spine density was observed after MCAO/R when counting the spine numbers. However, the decrease in the dendritic spine density was alleviated by agomir-212-5p (Fig. 7H-K). Together, these data indicated that miR-212-5p may play an important role in enhancing dendritic spine formation and in promoting dendritic growth.

### MiR-212-5p promotes neuroprotection by targeting PLXNA2 in vitro

**Fig. 8:**
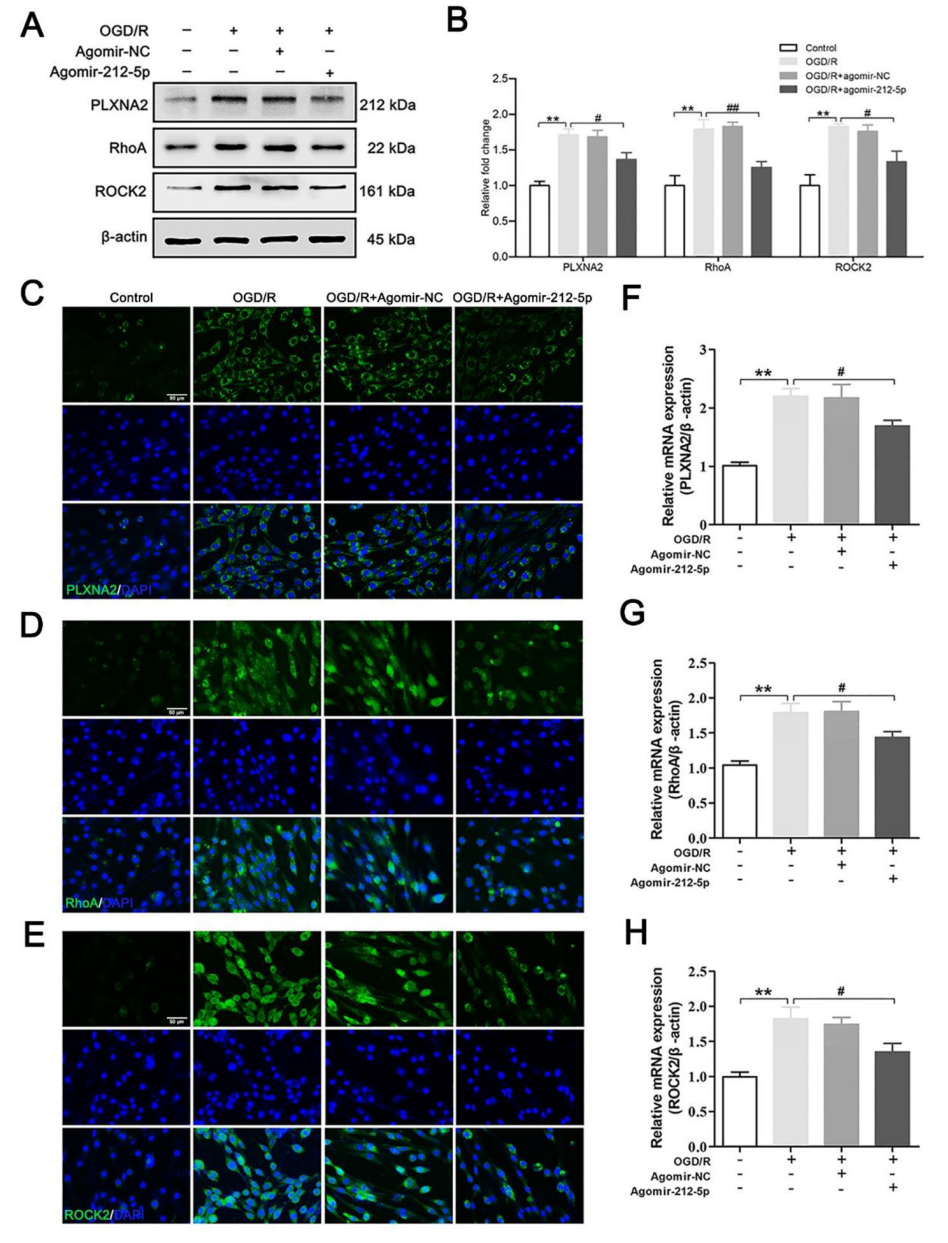
Agomir-212-5p suppressed the PLXNA2 and RhoA/ROCK2 in PC12 cells following OGD/R. **A** Representative photographs of western blots showing PLXNA2, RhoA and ROCK2 levels in each group. β-Actin was used as an internal reference. **B** Quantitative analysis of the western blot results. **C-E** Immunofluorescence staining results for the PLXNA2, RhoA and ROCK2 (in green) proteins in neurons in each group. Nuclei were stained with DAPI and are visualised in blue. Scale bar = 50 μm. **F-H** qRT–PCR was performed to assess the mRNA levels of PLXNA2, RhoA and ROCK2 in each group. The data are presented as the means ± SEM (n = 4–6 per group). *P < 0.05 and **P < 0.01 compared with the sham group, ^#^*P* < 0.05 and ^##^*P* < 0.01 compared with the MCAO/R group

For the in vitro study, PC12 cells exposed to an OGD environment were used to assess the neuroprotective effect of miR-212-5p. We first investigated the levels of the PLXNA2, RhoA and ROCK2 proteins using western blot analysis. The results for PLXNA2, RhoA and ROCK2 expression in cells were similar to those obtained in the rat brain tissue (Fig. 8A, B). Moreover, the intensities of PLXNA2, RhoA and ROCK2 immunofluorescence staining were increased after OGD/R injury, but the increases were reversed in the agomir-212-5p group (Fig. 8C-E). These results were further confirmed using qRT–PCR (Fig. 8F-H). Collectively, PLXNA2 may be involved in the effect of miR-212-5p.

### MiR-212-5p attenuates apoptosis and axonal degeneration in vitro

**Fig. 9:**
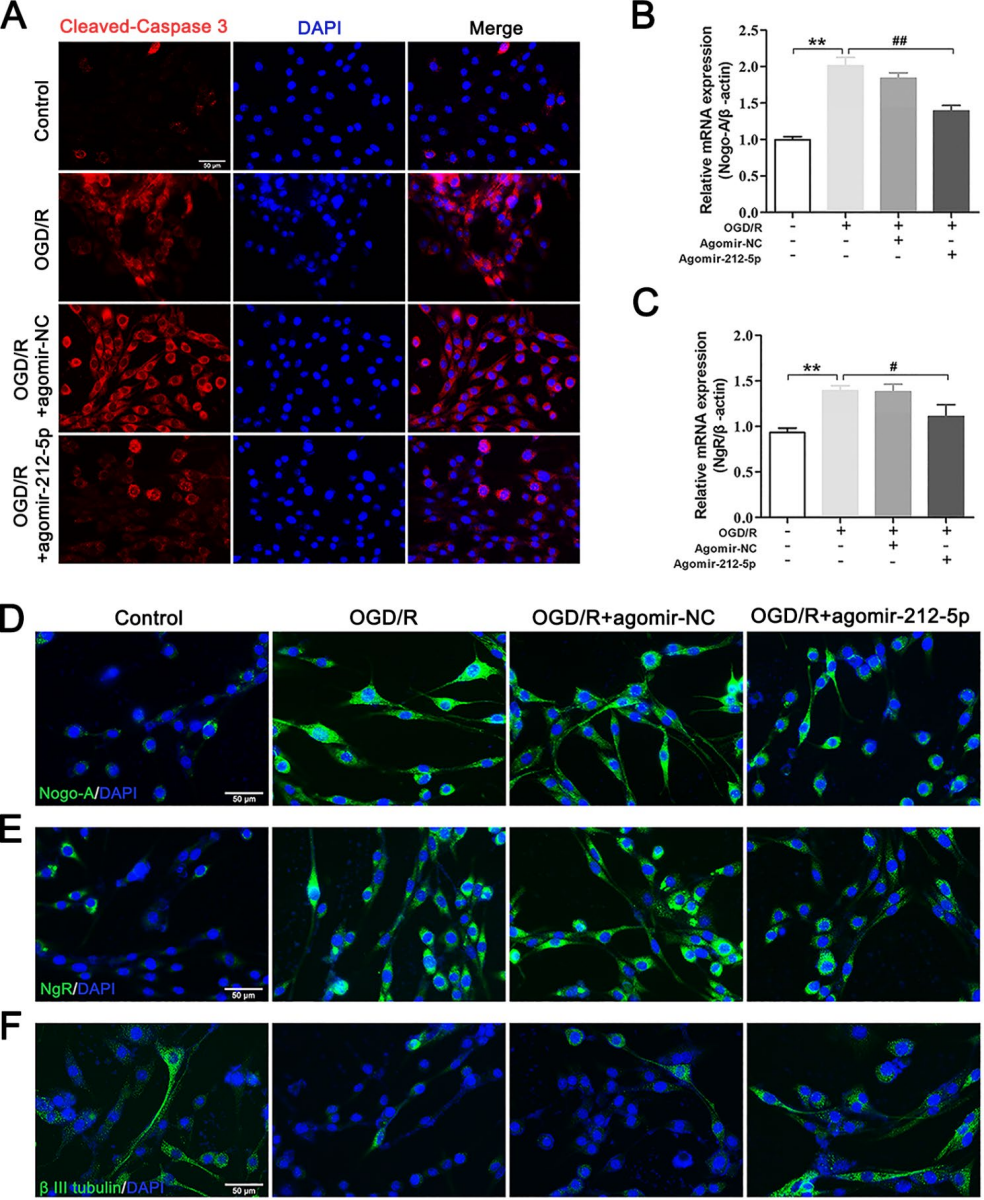
MiR-212-5p reduces neuronal apoptosis and attenuates axonal degeneration in PC12 cells following OGD/R. **A** Cleaved-Caspase 3 immunofluorescence staining (in green). Nuclei were stained with DAPI and are visualised in blue. Scale bar = 50 μm. **B, C** Nogo-A and NgR gene expression levels in PC12 cells. Gene expression levels were examined using qRT–PCR and normalised to β-actin. **D-F** Immunofluorescence staining was conducted to reveal the presence of Nogo-A, NgR and β III tubulin in PC12 cells. Nuclei were stained with DAPI and are visualised in blue. Scale bar = 50 μm. The data are presented as the means ± SEM (n = 5 per group). *P < 0.05 and **P < 0.01 compared with the sham group, ^#^*P* < 0.05 and ^##^*P* < 0.01 compared with the MCAO/R group

As shown in Fig. 9A, immunofluorescence staining showed a marked increase in Cleaved–Caspase 3 levels following OGD/R, whereas agomir-212-5p treatment significantly reduced cell death. The OGD/R group exhibited higher levels of the NogoA and NgR mRNAs than the agomir-212-5p treatment group (Fig. 9B, C). Similarly, cellular immunofluorescence experiments further confirmed these results. OGD/R increased Nogo-A and NgR mRNA expression and decreased β III tubulin expression, but agomir-212-5p treatment significantly reversed these changes (Fig. 9D-F). These results demonstrated that miR-212-5p exerts a neuroprotective effect on cell death in vitro.

## Discussion

M2 microglia have been reported to protect neurons through a paracrine mechanism or by releasing neurotrophic factors [24]. The mechanisms underlying the interaction of microglia with neurons for neuronal survival are poorly understood. Revealing the molecular mechanisms underlying M2 microglia’s protective role in ischemic stroke will contribute to identifying additional potential therapeutic targets for ischemic stroke. The current study is the first to show that a miR-212-5p intervention exerted a neuroprotective effect and ameliorated motor deficits in MCAO/R rats by targeting PLXNA2. This study reports several important discoveries. First, miR-212-5p expression was substantially decreased in microglial exosome extracts from the ischemic penumbra region of the ipsilateral hemisphere of the brain during the acute phase of ischemic stroke. Second, miR-212-5p treatment attenuated neuronal damage by restoring dendritic spine formation, dendritic growth, and synaptic plasticity and attenuating axon degeneration. Third, miR-212-5p ameliorates early brain injury and improves neurological performance and motor function in MCAO/R rats by directly targeting PLXNA2. Our results reveal that miR-212-5p may be a crucial factor contributing to neuroprotection that exerts its function by regulating the expression of its downstream target gene PLXNA2.

The early pathological changes of cerebral ischemia involve a series of complex reactions in the CNS, including genomic, molecular and cellular alterations, which may contribute substantially to neuronal damage [25]. Brain-resident immune cells such as astrocytes, microglia, or endothelial cells participate in activating inflammatory responses after ischemic injury and communicate with one another via the systemic production of inflammatory mediators and cytokines [25]. Microglia are first rapidly activated in response to brain injury, and activated microglia polarize to an anti-inflammatory (M2) phenotype during the early period following ischemic stroke and subsequently transform into a pro-inflammatory (M1) phenotype [26]. The neuroprotective effect of microglia on ischemic stroke primarily relies on microglia with the M2 phenotype [27]. M2 microglia exert neuroprotective effects by attenuating local inflammation, engulfing and clearing cell debris, and modulating tissue remodeling [28]. However, the mechanism underlying the effects of M2 microglia on neuronal functions still requires further clarification. A delayed neuronal death and excitotoxic injury in the brain’s ischemic penumbra are major markers of neuronal tissue damage following ischemic injury [29]. The surviving neurons in the ischemic penumbra directly accelerate neurological function recovery following ischemic stroke [30]. Thus, acute stroke treatment aims to salvage ischemic penumbra tissue in order to minimize long-term functional deficits.

Several miRNAs protect neurons from neuronal injury and exert neuroprotective effects. MiR-9 contributes to neuronal survival and regeneration after ischemia. Transfection with a miR-9 mimic increases the viability of neurons, facilitates neurite elongation and promotes neuronal proliferation, leading to functional recovery of ischemic stroke [31]. In a mouse ischemic stroke model, miR-215-associated neuroprotection suppresses autophagy activity and reduces apoptosis, which results in a further reduction in the infarct volume and improves functional recovery [32]. Upregulation of miR-21 reduces stroke-related inflammation, thereby protecting against BBB disruption, increasing neuronal cell survival, and importantly, enhancing functional outcomes following cerebral ischemia-reperfusion in rats [33]. According to recent studies, exosomes secreted from microglia are essential to mediate interactions between microglia and neurons by transferring proteins, mRNAs and miRNAs [34]. Exosomes miRNAs have a variety of functions, such as neural protection, apoptosis resistance, anti-oxidation, anti-inflammatory, and blood brain barrier repair [35]. Exosomal miR-124-3p is transported from microglial cells to neurons; furthermore, miR-124-3p regulates the Rela/ApoE pathway to ameliorate injury, which contributes to the recovery of cognitive outcomes [36]. In the current study, we found that miR-212-5p expression was significantly decreased in microglia exosomes following cerebral ischemia reperfusion using miRNA sequencing method. Thus, miR-212-5p may contribute to cerebral ischemia reperfusion and was chosen as a candidate miRNA for further study.

The miR-212 belongs to the miR-132/212 family, which having a high level of expression in the brain [37], and it is associated with neuronal remodeling, synaptic plasticity, and communication between immune cells and neurons [38, 39]. The expression of miR-132 is widespread throughout the CNS, with neurons having the highest level, followed by astrocytes, oligodendrocytes, and microglia, while miR-212 is predominantly expressed in non-neuronal cells [40]. MiR-132/212 contributes to neural development, maturation, morphogenesis and function. More importantly, miR-132/212 has a crucial role in regulating inflammation. It can reduce the infiltration of inflammatory cells, especially microglia, while reducing the release of inflammatory cytokines. Therefore, miR-212 is also called ‘neurimmiR’ since miR-212 participate in the cross-talk between neural and immune functions [41]. In our studies, M2 microglia were present at the highest levels on the third day after surgery, and miRNA sequencing results from exosomes extracted from microglial cells suggested a reduction in miR-212-5p expression. Our in vivo study revealed that miR-212-5p was downregulated in the ischemic penumbra region at 72 h after MCAO/R. The expression of miR-212-5p was also decreased in vitro after PC12 cells were subjected to OGD/R. Furthermore, identification of the miR-212-5p target genes is important for understanding the role of miR-212-5p in the pathological process of cerebral ischemia. In the current study, it was observed that PLXNA2 may be a direct target gene of miR-212-5p in cerebral ischemia.

PLXNA2 is a member of the PLXNs family, initially identified as important regulators of axon growth cone guidance and axon extension, which participates in axon repulsion during development [42]. According to a previous study, PLXNA2 limits axonal growth, which ultimately broadly limits recovery from CNS injury [43]. Over the past few years, PLXNs have been increasingly recognized as an important role in immunity, regulating various cellular signaling pathways in an independent or dependent manner [44]. Recent studies have revealed that PLXNs are important for maintaining microglial homeostasis, and they are closely related to inflammatory mediator production [45]. It has been reported that the crosstalk between PLXNA1 and Toll-like receptor 4 signaling pathway enhances the activation of microglia [46]. However, little is known about the mechanism of PLXNA2 in cerebral ischemia reperfusion-induced neural injury. Our previous studies found PLXNA2 to be an important mediator of inflammatory responses induced by microglia polarization after cerebral ischemia reperfusion in rats [47]. The findings suggested that PLXNA2 inhibition may be a potential mechanism associated with neural repair after acute stroke. In the current study, PLXNA2 expression in neurons in the ischemic penumbra region was inhibited by agomir-212-5p in MCAO/R rats. Based on these findings, there is a possibility that PLXNA2 is a downstream target gene for miR-212-5p that may play an important role in impaired functional recovery following ischemic stroke. Further, the PLXN family executes its functions by directly binding Rho family GTPases via an intracellular domain [48]. RhoA has been identified as a negative regulator of neurite outgrowth during development, and its major downstream effector, Rock2, is associated with the induction of RhoA-driven neurite retraction [49]. RhoA/Rock2 upregulation contributes to neuroinflammation, blood-brain barrier dysfunction, axon growth inhibition and neuronal apoptosis following ischemic stroke [50]. Inhibition of RhoA/Rock2 signalling may effectively induce axonal regeneration following injury [51]. It has been suggested that miR-30b facilitate neuronal regeneration and neurite growth by regulating the Sema3A/NRP-1/PlexinA1/RhoA/ROCK axis to promote functional recovery following spinal cord injury [52]. According to our study, the miR-212-5p agomir treatment also suppressed RhoA and ROCK2 expression in ischemic penumbra. Our data indicated that the administration of the miR-212-5p agomir enhances axonal protection after acute axonal injury, increases GAP-43 and β III tubulin protein expression and decreases Nogo-A and NgR expression. Moreover, the morphology of damaged dendrites was sufficiently restored.

This study still has several limitations. First, we mainly focused on the effect of miR-212-5p on the pathological progression of ischemic stroke and neuronal protection, while the effect of exosomes transfected with miR-212-5p requires further analysis. Second, we only validated the altered expression of this miRNA in the acute stage of MCAO/R. More studies are needed to explore the long-term effects of miR-212-5p.

## Conclusion

**Fig. 10:**
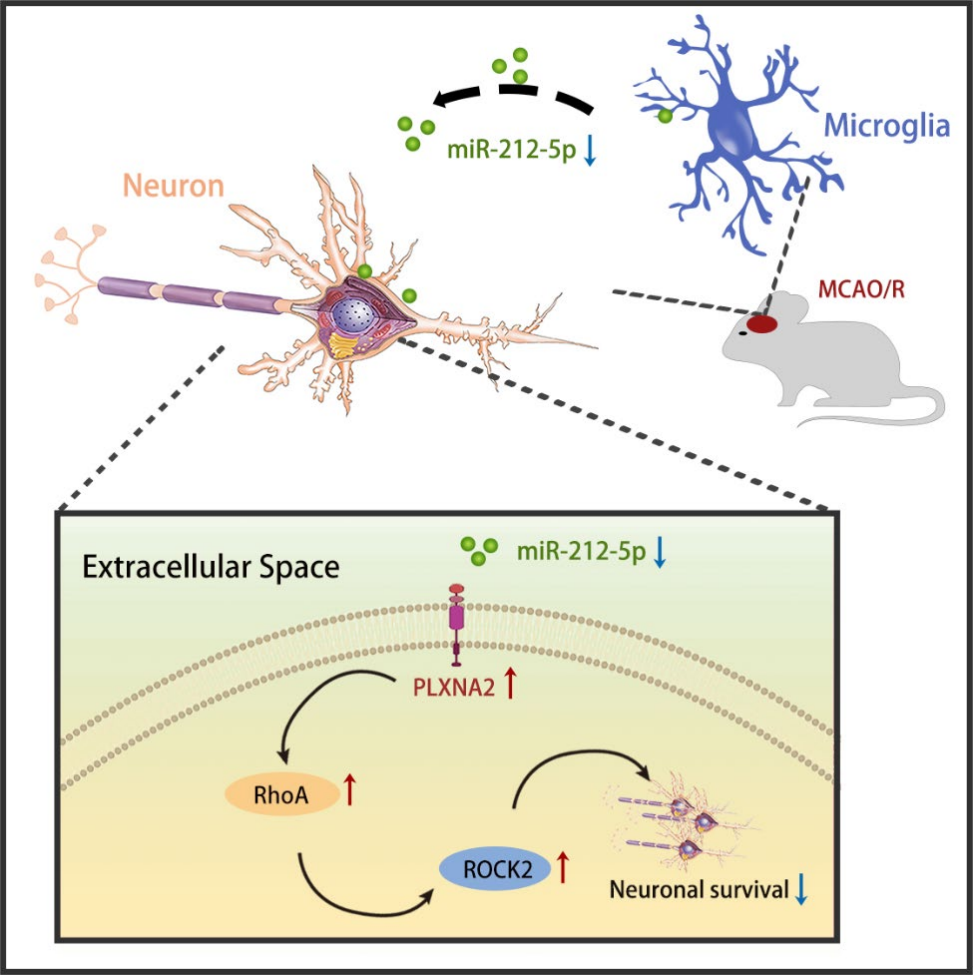
A schematic diagram showing the potential molecular mechanism by which the miR-212-5p agomir exerts a neuroprotective effect to alleviate ischemic neuronal damage

In conclusion, our findings reveal that PLXNA2 may be a target gene of miR-212-5p. MiR-212-5p and its target gene contribute to increase of neuronal survival, alleviation of axonal degeneration and improvement of motor function after MCAO/R (Fig. 10). Consequently, agomir-212-5p treatment may be a strategy for ischemic brain injury with great potential and promising clinical application value.

## Materials and methods

### Animals

We obtained adult male Sprague–Dawley (SD) rats weighing 260–280 g from Shanghai Laboratory Animal Research Center (Shanghai, China). Only male rats were used in this study because a previous study documented the neuroprotective effects of oestrogens on animal models of cerebral ischemia [53]. We housed all rats under standard laboratory conditions of of 23 ± 2 °C, 40–50% humidity, and 12 h–12 h light-dark cycle. Food and water were freely available to the animals. The Animal Ethics Committee of Shanghai University of Traditional Chinese Medicine reviewed and approved all experimental protocols, and all animal procedures followed the guidelines of the National Institutes of Health Guide for the Care and Use of Laboratory Animals.

### Animal model of ischemic stroke and intracerebroventricular injection

Pentobarbital sodium (30 mg/kg, intraperitoneal) was used to anesthetize rats. Then, rat was placed in a supine position, and a midline incision was made on the neck to expose the left external carotid artery (ECA), internal carotid artery (ICA) and common carotid artery (CCA). Surgical silk was used for ligation of the ECA to block blood flow, and the left ICA and CCA were temporarily clamped using microvascular clips. In the ECA, a small incision was made. Then, a silicon-coated suture (L3600, Jia Ling Biotechnology Co. Ltd., Guangzhou, China) was gently advanced from the ECA stump into the ICA to occlude blood flow in the MCA. The silicon-coated monofilament nylon suture was fixed for 2 h, and ICA perfusion was restored. Rats in the sham group underwent the same operation with the exception of the monofilament nylon suture being inserted.

The animals were randomly assigned to 4 groups: the sham group, MCAO/R group, MCAO/R + agomir-NC group and MCAO/R + agomir-212-5p group. According to previous reports, agomir-212-5p and agomir-NC were injected intracerebroventricular (ICV) injection 30 min before MCAO/R [54]. The skull was exposed by making an incision along the midline of the scalp. Injection coordinates were 1.0 mm posterior and 1.5 mm lateral to the bregma at a depth of 3.5 mm. Agomir-212-5p or agomir-NC (10 µM in 7 µl) was slowly injected. Simultaneously, 7 µl of 0.9% NaCl was injected into lateral ventricle both in the sham and MCAO/R groups. The needle was retained in situ for 5 min following injection.

### Isolation of microglial exosomes from the injured brain

Microglial exosomes from the ischemic penumbra portion of the cortex were isolated as previously described [36]. In each group, the ischemic penumbra portion of the cortex was rapidly isolated from the left hemisphere. Samples were digested with collagenase type 3 (75 U/ml, Worthington Biochemical Corporation, Lakewood, NJ, USA) at 37 °C for 15 min. Then, the cells were centrifuged at 2000 × g at 4 °C for 10 min to remove dead cells and subsequently centrifuged again for 30 min at 10,000 × g at 4 °C to remove cellular debris. After collecting the supernatant, it was filtered through a 0.22 μm filter. Samples were centrifuged at 100,000 × g at 4 °C for 70 min, and the supernatant was removed. Calcium- and magnesium-free Dulbecco’s PBS (Gibco, USA) was applied to resuspend the exosome pellet. Samples were incubated for 1 h with 50 mL of 3% BSA containing 1.5 µg of anti-CD11b biotinylated antibody (NB110-89474B, Novus, CO, USA) at room temperature to isolate microglial exosomes. Subsequently, the mixture was incubated with Pierce Streptavidin Plus UltraLink Resin (Thermo Fisher Scientific, Waltham, MA, USA) at room temperature for 30 min. Thereafter, centrifuged at 800 × g for 10 min at 4 °C of the samples was performed. The supernatant was discarded after centrifugation, and microglial exosomes were recovered and stored at 4 °C until use in subsequent analysis.

### Exosome identification

Nanoparticle tracking analysis (NTA), transmission electron microscopy (TEM) and western blot analysis were applied to characterize exosomes. First, exosomes were analysed by performing NTA. In order to measure the size and concentration of exosome particles, we used NTA ZetaView PMX 110 (Particle Metrix, Meerbusch, Germany) and ZetaView 8.04.02. Isolated exosome samples were appropriately diluted in 1 × PBS (Biological Industries, Israel). NTA was performed at 11 positions, and the measurement results were recorded. The ZetaView system was calibrated using 110 nm polystyrene particles. Temperature was maintained at approximately 26.26 and 27.21 °C.

Exosome morphology was analysed using negative-staining TEM. In 50 µl of 2% PFA, exosomes were resuspended, and the exosomal suspension (5 µl) was placed on Formvar/carbon-loaded copper mesh and washed 3 times in PBS. The copper mesh was floated onto a drop of 1% glutaraldehyde for 5 min and then washed. After washes with distilled water, copper meshes were placed on droplets of a uranyl-oxalate solution for 5 min and then transferred to methylcellulose for 10 min. Excess liquid was removed with filter paper and samples were allowed to air dry for 10 min. Electron micrographs were obtained with a JEOL 1230 transmission electron microscope (JEOL, Tokyo, Japan).

The expression of the exosome marker proteins CD9, CD63 and CD81 was evaluated using western blot analysis. To identify microglia-derived exosomes, CD11b was used. Exosome samples were first lysed using RIPA buffer, and total protein was separated on 12% SDS–PAGE gels and transferred to a polyvinylidene difluoride (PVDF) membrane (Millipore, Billerica, MA, USA). After blocking the membranes for 1 h with 10% skim milk formulated in Tris-HCI buffered saline solution (TBST), they were overnight incubated with primary antibodies. Antibodies included CD9 (1:1000, ab92726, Abcam, Cambridge, UK), CD63 (1:1000, AF5117, Affinity Biosciences, Cincinnati, OH, USA), CD81 (1:500, DF2306, Affinity Biosciences, Cincinnati, OH, USA) and ITGAM/CD11b (1:500, DF2911, Affinity Biosciences, Cincinnati, OH, USA). The PVDF membranes were then incubated with HRP-conjugated secondary antibodies (1:5000) at room temperature for 1 h. UVP BioSpectrum imaging system (BioSpectrum 410, USA) was used to detect protein signals using WesternBright ECL reagent (Advansta, USA).

### MiRNA sequencing analysis and target gene prediction

MiRNA sequencing was performed by KangCheng Biotech, Shanghai, China. After the total RNA samples were extracted with TRIzol reagent and tested and quantified using agarose gel electrophoresis and a NanoDrop spectrophotometer, the library was constructed, and the quality of the library was tested using an Agilent 2100 Bioanalyzer. In order to generate single-stranded DNA, mixed sequencing libraries of different samples were denatured with 0.1 M NaOH, and 51 cycles of sequencing were performed using an Illumina NextSeq 500 sequencer according to the supplier’s instructions. TargetScan (http://targetscan.org/) and miRDB (http://mirdb.org/miRDB/) were used to predict miR-212-5p downstream target genes, and genes related to neurodegeneration were selected for further analysis.

### Dual-luciferase assay

The wild-type PLXNA2 3′UTR (WT) or PLXNA2 3′UTR mutant (MUT) of the miR-212-5p binding site, was inserted into the dual-luciferase pmirGLO vector (GenePharma, Shanghai, China). The WT and MUT luciferase reporter plasmids were cotransfected into PC12 cells along with miR-212-5p or negative control (NC) using Lipofectamine 2000 (GenePharma Co., Ltd., Shanghai, China) according to the manufacturer’s protocol. Luminescence was detected with a dual-luciferase reporter assay (Promega, Madison, WI, USA) using a GloMax instrument (Promega).

### Cell culture, treatments, and oxygen–glucose deprivation/reperfusion (OGD/R)

We obtained PC12 cells from the Shanghai Institute of Cell Biology and cultured them in DMEM containing 10% FBS and 1% penicillin/streptomycin. The cells were cultured at 37 °C in a humidified 5% CO_2_ environment. For experiments with transfected cells, cells were transfected with 100 nM agomir-212-5p or agomir-NC. Transfected cells were used for subsequent experiments 24 h after transfection. We then changed the culture medium to glucose-free culture medium. The cells were incubated at 37 °C in a low-oxygen incubator for 4 h (1% O_2_, 94% N_2_, and 5% CO_2_). Subsequently, the cells were switched to regular medium and maintained under standard (5% CO_2_ and 37 °C) conditions for reperfusion.

### Behavioral tests

Neurological deficits were scored by an observer blinded to the groups using Zea Longa scores [55]: 0 = no symptoms of neurological deficits; 1 = cannot extend the right forepaw completely; 2 = circling to the right; 3 = falling to the right; 4 = unable to walk spontaneously.

The foot fault test was performed in accordance with a previously described method [56]. Eight rats were included in each group. We tested rats’ ability to walk over a ladder with irregular spacing (1–3 cm) on 3 occasions and video recorded to assess the impairment in right forelimb function after stroke. Scoring was based on the following criteria: 0 = total miss; 1 = deep slip; 2 = slight slip; 3 = replacement; 4 = correction; 5 = partial placement; 6 = correct placement. The misstep rate refers to the number of steps with a walking score of 0, 1 or 2. To calculate the misstep rate, we used the following formula: (the number of wrong steps of the right forelimb/total steps) × 100%.

The motor performance and coordination of animals were evaluated using an automated quantitative gait analysis system (CatWalk™, Wageningen, The Netherlands). Each group included eight rats. During the experiment, the room was dark and quiet. Prior to experimentation, each rat performed 3 trials without any interruption to cross the pressure-sensitive plate of the Catwalk system. The rat was placed at one end of a 150-cm-long runway consisting of a glass platform covered by a black tunnel with a food reward at the opposite end of the runway. This test was performed before and on days 1, 3, 5, and 7 following the surgery, respectively. Data were acquired and analysed using CatWalk version 10.6 software.

### Motor evoked potential (MEP)

Electrophysiological tests were conducted on the 7th day following modelling with an electromyography evoked potentiometer (9033A07, Keypoint; Medronic, Skovlunde, Denmark). After the rat was anesthetized, the recording electrode was placed on the right biceps brachii, and the stimulating electrode was inserted into the rat’s upper jaw which was near the left motor cortex. Single square-wave electrical pulse (100 µs) was applied, and the stimulation intensity increased gradually until latency and amplitude were no longer changed. The latency and amplitude of the MEP were obtained.

### Resting-state functional magnetic resonance imaging (fMRI)

We used a 11.7 T scanner (Bruker, Ettlingen, Germany) for the fMRI scans. 5% isoflurane was used to induce anesthesia, and anesthesia was maintained with isoflurane (1.5%) and dexmedetomidine (0.05 mg/kg). Resting-state fMRI data were collected using single echo planar imaging (EPI) with the following parameters: flip angle = 90°, slice thickness = 0.3 mm, number of average = 1, repetition time (TR) = 3000ms, Echo time (TE) = 8.142 ms, field of vision (FOV) = 27 × 27 mm^2^. The fMRI data were preprocessed using Statistical Parametric Mapping 8 (SPM 8) toolbox (http://www.fil.ion.ucl.ac.uk/spm/) based on the MATLAB (R2014b; Mathworks, Natick, MA, USA). All the images were transformed to Nifti format, followed by slice timing and realign. Non-brain tissues were removed manually and each image was manually reoriented by setting the origin to the anterior commissure. Finally, images processing with normalize and smooth. In the following FC analysis, the ipsilateral (left) motor cortex was selected as the region of interest (ROI) to examine whole-brain functional connectivity, and then performing Fisher’s Z transformation. The comparison between groups was carried out using two-sample t-test in SPM8. *P* < 0.001 was considered to be statistically significant.

### Evaluation of the infarct volume

Rat brains were quickly removed and frozen at -20 °C for 20 min; subsequently, the frozen brains were coronally sliced into five 2-mm-thick coronal slices. Brain tissue slices were stained with 2% 2,3,5-triphenyltetrazolium chloride (TTC) (Sigma-Aldrich, St. Louis, MO, USA) and incubated at 37 °C for 20 min in the dark, followed by fixation with 4% paraformaldehyde (PFA). The infarct area was calculated using Image J software.

### Western blot analysis

Brain tissue corresponding to the ischemic penumbra and cells were collected and homogenised with RIPA lysis buffer (Beyotime, Shanghai, China). By using BCA protein assay reagents (Beyotime, Shanghai, China), protein concentrations were determined. A tissue sample (80 µg) and cellular sample (30 µg) were loaded and separated on SDS–PAGE gels prior to being transferred to a PVDF membrane. The PVDF membranes were blocked with protein-free rapid blocking buffer for 20 min and then incubated with primary antibodies against PLXNA2 (1:1000, #5658, Cell Signaling Technology, Beverly, MA, USA), Rho protein A (RhoA) (1:5000, ab187027, Abcam, Cambridge, UK), Rho-associated kinases 2 (ROCK2) (1:10000, ab125025, Abcam, Cambridge, UK), GAP43 (1:100000, ab75810, Abcam, Cambridge, UK), neurite outgrowth inhibitor-A (Nogo-A, 1:1000, DF8581, Affinity Biosciences, Cincinnati, OH, USA), nogo receptor (NgR, 1:500 DF13593, Affinity Biosciences, Cincinnati, OH, USA), and β-actin (1:1000, #4970, Cell Signaling Technology, Beverly, MA, USA) at 4 °C overnight. On the following day, the membranes were incubated with HRP-conjugated secondary antibodies (1:5000) for 1 h at room temperature. The UVP BioSpectrum imaging system (BioSpectrum 410, USA) was used in conjunction with WesternBright ECL reagent (Advansta, USA) in order to detect protein signals. Finally, all bands were subjected to densitometry analysis with ImageJ.

### H&E and nissl staining

For Nissl staining, haematoxylin–eosin (H&E) staining and immunofluorescence staining, samples of brain were fixated in 4% paraformaldehyde, then dehydrated and embedded in paraffin. Subsequently, brain Sect. (5 μm thick) were fixed on poly-L-lysine-coated slides for H&E staining and Nissl staining. For HE staining, the slices were stained with haematoxylin and eosin with an H&E assay kit (G1003, Servicebio, Wuhan, China). Slices were stained with 0.1% cresyl violet for Nissl staining (G1036, Servicebio).

### Immunofluorescence staining

For paraffin tissue sections, sections were dewaxed with xylene and rehydrated with alcohol. The sections were subjected to heat-induced antigen retrieval, blocked with 10% goat serum albumin containing 0.3% Triton X-100, and incubated with the indicated primary antibodies overnight at 4°C. PC12 cells were washed with PBS, fixed with 4% paraformaldehyde, permeabilized with 0.3% Triton X-100 in PBS for 15 min, and incubated with 10% goat serum albumin at room temperature for 1 h. Next, slides were incubated at 4°C overnight with primary antibodies against Iba1 (1:100, ab15690, Abcam, Cambridge, UK), CD206 (1:200, ab64693,, Abcam, Cambridge, UK), CD86 (1:200, PA5-88284, ThermoFisher, Waltham, MA, USA), PLXNA2 (1:200), RhoA (1:150), ROCK2 (1:200), NeuN (1:200, ab104224, Abcam, Cambridge, UK), Cleaved-Caspase 3 (1:200, BF0711, Affinity Biosciences, Cincinnati, OH, USA), Nogo-A (1:200), NgR (1:200), microtubule-associated protein 2 (MAP-2, 1:200, 17490-1-AP, Proteintech, Wuhan, China) and β III tubulin (1:500, ab52623, Abcam, Cambridge, UK). The following day, the samples were incubated with Alexa Fluor® 488-conjugated AffiniPure goat anti-rabbit (1:200, 33106ES60, YEASEN, Shanghai, China) or Alexa Fluor® 594-conjugated AffiniPure goat anti-mouse (1:200, 33212ES60, YEASEN, Shanghai, China) secondary antibodies at 37°C for 30 min. Nuclear staining was performed using 4’, 6-Diamidino-2-phenylindole (DAPI, Beyotime, Shanghai, China). All images of immunofluorescence staining were captured using a fluorescence microscope (DM6000B, Leica, Germany). Images were analysed using ImageJ software.

### Quantitative RT–PCR (qRT–PCR)

Total RNA samples from cells and brain tissues using TRIzol reagent (Invitrogen, Carlsbad, CA, USA) and were reversely transcribed to cDNA using reverse transcription kit (A3500, Promega, Madison, WI, USA). Target genes were examined using a SYBR-Green RT–PCR kit (QPK-212, TOYOBO, Osaka, Japan) and a LightCycler 480 system (Roche, San Francisco, CA, USA). PCR conditions were as follows: predenaturation for 5 min at 95 °C, followed by 40 cycles of 95 °C for 10 s, 60 °C for 10 s, and 72 °C for 10 s. U6 (for miRNA) and β-actin (for mRNA) were used as an internal inference. Reactions were performed in triplicate, and the 2^–ΔΔCt^ method was applied to estimate the results [57]. Tables 2 and 3 show the primer sequences designed for this study.

**Table 2:** Primers sequence for quantitative real-time Polymerase Chain Reaction

Gene	Forward primer (5′-3′)	Reverse primer (5′-3′)
*miR-30c-5p*	GCGCGTGTAAACATCCTACACT	AGTGCAGGGTCCGAGGTATT
*miR-126a-5p*	GCGCGCATTATTACTTTTGG	AGTGCAGGGTCCGAGGTATT
*miR-128-3p*	CGCGTCACAGTGAACCGGT	AGTGCAGGGTCCGAGGTATT
*miR-212-5p*	GCGACCTTGGCTCTAGACTGC	AGTGCAGGGTCCGAGGTATT
*miR-1949*	CGCGTATACCAGGATGTCAGC	AGTGCAGGGTCCGAGGTATT
*U6*	AGAGAAGATTAGCATGGCCCCTG	ATCCAGTGCAGGGTCCGAGG

**Table 3:** Results of functional connectivity analysis among four groups

Contrast				MNI Coordinates		
Name	Region Label	Extent	t-value	x	y	z
S > M	R_Cortex_Retrosplenial	36	5.634	12	32	13
	L_Cortex_Somatosensory	23	5.114	-36	20	-53
	R_Caudate_Putamen	16	4.766	26	-40	-53
	R_Hippocampus_Ventral	10	4.473	53	-28	11
	L_Caudate_Putamen	12	4.210	-24	-9	-57
S > M + NC	L_Cortex_Motor	23	5.152	-26	22	-85
	L_Cortex_Medial_Prefrontal	14	4.971	-9	15	-95
	R_Caudate_Putamen	16	4.676	36	-30	-49
M > M + agomir	R_Olfactory_Nuclei	28	5.449	14	-13	-95
M < M + agomir	R_Cortex_Somatosensory	15	-6.398	38	28	-39
	R_Cortex_Motor	12	-4.946	7	38	-27
	L_Cortex_Piriform	10	-4.824	-38	-34	-57
M + NC > M + agomir	R_Amygdala	13	4.492	51	-49	-21
M + NC < M + agomir	L_Cortex_Motor	37	-5.958	-24	18	-83
	R_Periaqueductal_Grey	10	-4.569	5	-18	19

Abbreviations: S, Sham group; M, MCAO/R group; M+NC, MCAO/R+agomir-NC group; M+agomir, MCAO/R+agomir-212-5p group; R, right; L, left.

### Transmission electron microscopy

After cutting brain tissues into 1mm^3^ pieces, we fixed them with 2.5% glutaraldehyde for 2 h. The tissues were then washed and fixed in osmic acid (1%) for 1 h, dehydrated in ethanol, and embedded. Then, 50-nm ultrathin sections were prepared and stained with uranyl acetate and lead citrate. The images were captured using a transmission electron microscope (Tecnai G2 Spirit Bio TWIN, FEI Company, USA).

### Golgi-Cox staining, sholl analysis and measurement of the spine density

The rat brain was dissected and subjected to Golgi-Cox staining with an FD Rapid GolgiStain Kit (FD Neurotechnologies, Columbia, MD). Rats were deeply anaesthetized with sodium pentobarbital, and brains were removed as quickly as possible while handling carefully avoiding damage to the brain tissue. Briefly, the extracted brains were soaked in a mixture of a 1:1 volumetric ratio of solutions A:B for 2 weeks in the dark at room temperature. Then, the brain tissue was transferred into another solution (C) and stored in the dark for 3 days. Coronal slices (100 μm thickness) were prepared using a vibrating slicer (Leica, VT1200 S) and stained using standard staining procedures. For the Sholl analysis, NeuronJ plugin was used for neuronal tracing and Sholl Analysis plugin (http://fiji.sc/Sholl_Analysis) was used in ImageJ. The cell body was selected, and a count of dendrite intersections around the center of the cell body was performed at 20-µm intervals. The number of spines on segments of 10 μm dendrites was counted to evaluate the dendritic spine density.

### Statistical analysis

An analysis of the data was performed with SPSS Statistics software (version 22; SPSS, Chicago, IL, USA). We presented our data as mean ± standard errors of the means (SEM). The independent sample t test was used to detect significant differences between two groups. For comparisons between multiple groups, one-way analysis of variance (ANOVA) was performed. An additional post-hoc comparison was made using the least significant difference (LSD) test in the case of equal variances and Dunnett’s T3 in the case of unequal variances. It was considered statistically significant when the *P* was less than 0.05.

## Data Availability

The datasets used and/or analyzed during the current study are available from the corresponding author on reasonable request.

## Abbreviations

MCAO/R: Middle cerebral artery occlusion /reperfusion
OGD/R: Oxygen-glucose deprivation (OGD/R) and reperfusion
CCA: Common carotid artery
ICA: Internal carotid artery
ECA: External carotid artery
SD rat: Sprague-Dawley rat
Iba-1: Microglia
PLXNA2: Plexin A2
GAP-43: Growth associated protein-43
IL-1β: Interleukin-1β
IL-10: Interleukin-10
IL-6: Interleukin-6
INOS: Inducible nitric oxide synthase
MEP: Motor evoked potentials
Nogo-A: Neurite outgrowth inhibitor-A
NTA: Nanoparticle tracking analysis
RF: Right forelimb
RH: Right hindlimb
RhoA: Ras homolog family member A
ROCK2: Rho-associated coiled-coil forming protein kinase 2
TTC: 2,3,5-trihenyl tetrazolium chloride
H&E: Hematoxylin and eosin
TEM: Transmission electron microscopy
FMRI: Functional magnetic resonance imaging
